# GOPhage: protein function annotation for bacteriophages by integrating the genomic context

**DOI:** 10.1093/bib/bbaf014

**Published:** 2025-01-22

**Authors:** Jiaojiao Guan, Yongxin Ji, Cheng Peng, Wei Zou, Xubo Tang, Jiayu Shang, Yanni Sun

**Affiliations:** Department of Electrical Engineering, City University of Hong Kong, 83 Tat Chee Ave, Kowloon Tong, Hong Kong (SAR), China; Department of Electrical Engineering, City University of Hong Kong, 83 Tat Chee Ave, Kowloon Tong, Hong Kong (SAR), China; Department of Electrical Engineering, City University of Hong Kong, 83 Tat Chee Ave, Kowloon Tong, Hong Kong (SAR), China; Department of Electrical Engineering, City University of Hong Kong, 83 Tat Chee Ave, Kowloon Tong, Hong Kong (SAR), China; Department of Electrical Engineering, City University of Hong Kong, 83 Tat Chee Ave, Kowloon Tong, Hong Kong (SAR), China; Department of Information Engineering, Chinese University of Hong Kong, Shatin, New Territories, Hong Kong (SAR), China; Department of Electrical Engineering, City University of Hong Kong, 83 Tat Chee Ave, Kowloon Tong, Hong Kong (SAR), China

**Keywords:** bacteriophages, protein function annotation, protein large language model, genomic contextual information

## Abstract

Bacteriophages are viruses that target bacteria, playing a crucial role in microbial ecology. Phage proteins are important in understanding phage biology, such as virus infection, replication, and evolution. Although a large number of new phages have been identified via metagenomic sequencing, many of them have limited protein function annotation. Accurate function annotation of phage proteins presents several challenges, including their inherent diversity and the scarcity of annotated ones. Existing tools have yet to fully leverage the unique properties of phages in annotating protein functions. In this work, we propose a new protein function annotation tool for phages by leveraging the modular genomic structure of phage genomes. By employing embeddings from the latest protein foundation models and Transformer to capture contextual information between proteins in phage genomes, GOPhage surpasses state-of-the-art methods in annotating diverged proteins and proteins with uncommon functions by 6.78% and 13.05% improvement, respectively. GOPhage can annotate proteins lacking homology search results, which is critical for characterizing the rapidly accumulating phage genomes. We demonstrate the utility of GOPhage by identifying 688 potential holins in phages, which exhibit high structural conservation with known holins. The results show the potential of GOPhage to extend our understanding of newly discovered phages.

## Introduction

Bacteriophages (phages) are viruses that can infect bacterial cells. They are highly prevalent and abundant in the biosphere, being found in various environmental matrices, including gastrointestinal tracts of animals, water bodies, and soil [1–3]. Accumulating studies have demonstrated the important role of phages in microbial communities. For example, phages have been observed to facilitate the horizontal transfer of genes between bacteria, which can influence bacterial adaptation, evolution, and acquisition of new functionalities [4]. In addition, they can modulate the abundance and diversity of bacterial populations by killing their host [5]. Due to the increasing threats posed by antibiotic resistance, phages have gained significant attention as potential alternatives to traditional antibiotics, as they can lyse pathogenic bacteria [6–8].

Despite the significance of phages, the efficacy of their applications heavily relies on prior knowledge of protein functions. Understanding the protein function enables us to identify phage proteins that can target and disrupt essential bacterial processes, offering the potential for the development of targeted antimicrobial therapies [9]. For example, holin proteins, known for their cell-killing capabilities and broad host range, have gained significant attention for their potential applications in bacterial control [10, 11]. To accelerate the application of phages, it is crucial to figure out the annotation of the proteins in phages.

Gene Ontology (GO) terms are widely used to annotate the phage proteins. They are standardized vocabulary and hierarchical frameworks comprising three key dimensions: biological process (BP), cellular component (CC), and molecular function (MF) [12]. BP encompasses the sequences of events or pathways in which proteins participate, such as cellular signaling or metabolic processes, while CC pertains to the subcellular locations or structures where proteins are localized, such as the nucleus or plasma membrane. The MF aspect centers on the distinct activities and tasks carried out by proteins, such as enzyme catalysis or receptor binding.

However, there are two major challenges to using GO terms to annotate phage protein. First, the number of phage proteins with known GO labels is limited. Until 27 February 2024, the total number of phage proteins from the National Center for Biotechnology Information Reference Sequence Database (NCBI RefSeq) is $541\,060$, derived from $5160$ complete genomes. However, only 20.85% percent of proteins have GO labels. This scarcity of labeled proteins results in an insufficient database for comprehensive functional annotation. Second, although phage encodes a small number of proteins compared with their hosts, these proteins exhibit a remarkable degree of functional diversity. For example, among the 1173 phage proteins provided by the UniProtKB database, there are a total of 912 GO terms. This means that on average each GO label contains less than two supporting samples and will bring challenges to computational methods. Moreover, the distribution of these GO terms is imbalanced, with certain terms being more prevalent or specific than others. This imbalanced label distribution poses a significant challenge to accurate classification. These obstacles impose great requirements on annotation tools.

Several attempts have been made to analyze and annotate protein functions. They can be categorized into two types: homology-based and deep learning-based methods. The summarized information of the state-of-art methods is listed in Table 1. Homology-based methods, such as DiamondScore [13] and DiamondBlast [13], rely on sequence similarity to infer protein function. These methods assume that proteins with similar sequences share similar functions. However, due to the extensive genetic diversity and rapid evolution of phages, phage proteins may not have a significant sequence similarity when aligned to the reference database.

**Table 1:** The introduction of recent protein function annotation tools, with “DL” referring to deep learning-based solutions.

Tool	Type	Method
DiamondScore	Homology	DIAMOND BLASTP
DiamondBlast	Homology	BLAST
DeepGOCNN	DL	CNN
DeepGOPlus	Hybrid	CNN + DIAMOND BLASTP
ATGO	DL	ESM-1b + Triplet neural network
PFresGO	DL	ProtT5 + GO term relationship
NetGO3.0	DL	ESM-1b + Logistic regression
GPSFun	DL	ESMFold + ProtTrans + GNN
DeepGO-SE	DL	ESM2 + Semantic entailment

To annotate more proteins, most deep-learning methods formulate protein function annotation as a multi-label prediction task, where protein sequences or extracted features are used as the model input, and the predicted GO terms represent outputs. For example, DeepGOPlus leverages convolutional neural networks (CNNs) to make annotations based solely on sequence information, and it combines these predictions with alignment-based searches [13]. ATGO [14] utilizes the ESM-1b large language model to extract protein sequence embeddings, enhancing similarity among functions through a triplet network. In contrast, PFresGO [15] incorporates the hierarchical relationships of GO terms using Anc2Vec [16] and the ProtT5 model for embedding extraction, employing a cross-attention mechanism to improve annotation accuracy. NetGO 3.0 [17] replaces the Seq-RNN module of NetGO 2.0 [18] with ESM-1b and logistic regression, integrating multiple data sources, including protein sequences and GO term frequencies. GPSFun [19] employs graph neural networks to learn 3D structural features predicted by ESMFold [20], while DeepGO-SE [21] utilizes the ESM2 [20] model to generate approximate GO models, with a neural network predicting function statements. However, the primary information available for phage proteins is often limited to the protein sequence, with restricted access to additional data such as protein interactions or literature references. In addition, the methods described previously overlook the unique properties specific to phage proteins. Thus, there is considerable potential for enhancing the annotation of phage proteins. In our investigation, we have discovered that the order of phage protein functions exhibits a high level of conservation within the same genus. It means that the proteins in the surrounding context can provide valuable insights for predicting protein functions in phages.

In this work, we present a novel method, GOPhage, for phage protein annotation by integrating the powerful foundation model with the unique properties of phages. There are two main steps in our GOPhage framework. First, we utilize a pre-trained protein language model (PLM), ESM2 [20], to encode phage proteins. ESM2 has acquired a comprehensive understanding of various protein features, including aspects such as 3D structure and interaction relationships during training. Thus, it can effectively return meaningful representations for phage proteins. Second, we reformat the phage genomes into protein sentences using embeddings obtained from the PLM. Then, we train a Transformer-based natural language model to learn and leverage inherent order association among phage proteins. By considering the positions of proteins and their functions within the genomic context, the model is expected to further improve phage protein annotation. The experiments demonstrated a significant advantage of GOPhage in accurately predicting GO terms, achieving impressive area under the precision-recall curve (AUPR) scores of 0.8636, 0.8882, and 0.8277 for BP, CC, and MF ontology, respectively. Notably, GOPhage showcased substantial improvements in predicting the functions of proteins that lacked alignment with the database and minority GO labels, addressing an important challenge in functional annotation. In the case study, GOPhage demonstrates great promise in unraveling the functions of key phage proteins that lack alignment with the reference database. We identified 688 holin proteins and showed prediction reliability based on structural homology. Thus, GOPhage has the potential to accelerate and enhance the comprehensive understanding of phages and their biological processes.

## Methods and materials

**Figure 1:**
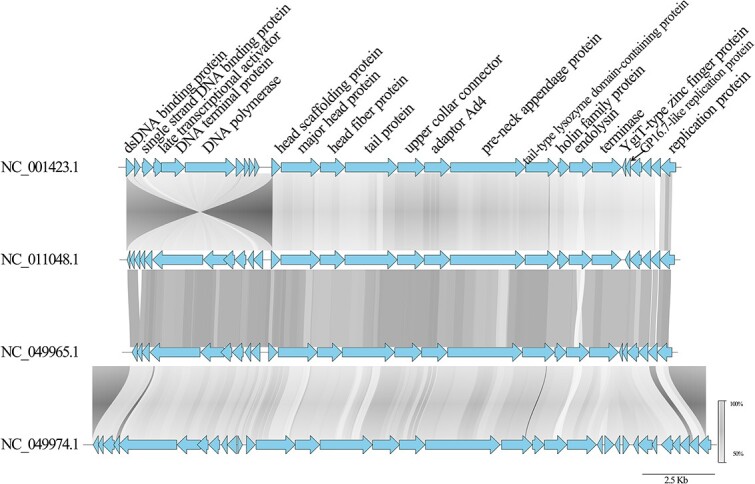
The function order of proteins within four phage genomes, where blue arrow represents the protein and the gray link shows the similarity among proteins.

The proteins in the phage sequences are similar to the words in the natural language. Thus, the phage genomes can be viewed as a language of phage life that exhibits distinct features. One notable observation of these phage languages is that phage proteins within the same genus tend to maintain a consistent arrangement. For instance, Fig. 1 reveals a distinct pattern in the order of proteins within the *Salasvirus* genus. These characteristics inspire us to reformat the phage genomes into sentences with contextual proteins and predict the annotations based on the surrounding information. In the following section, we will detail how GOPhage leverages the contextual information for phage protein annotation.

### Embedding protein sequences

**Figure 2:**
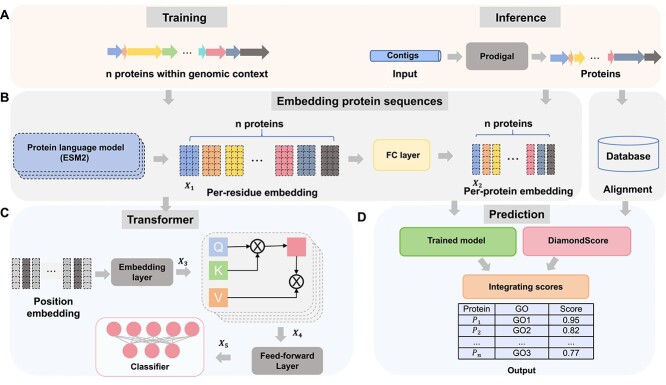
The architecture of GOPhage, including data processing steps for training and inference in (A), leveraging the ESM2 model in (B) for per-residue embeddings, utilizing the Transformer model in (C) for contextual relationships, and integrating alignment-based methods in (D) to produce Gene Ontology (GO) term prediction scores.

Figure 2 shows the architecture of the GOPhage model. In Fig. 2A, let the number of proteins of a phage genome in the training process be $n$. The first step is to encode the phage genomes by generating the embedding of the $n$ proteins. To obtain protein embedding, we employ the ESM2 model, which is pre-trained on protein sequences sourced from UR50/D. During training, ESM2 selects 15% amino acids for masking and predicts amino acids at the masked position. Based on a third-party benchmark result [22], ESM2-33 performs better than the ProtT5 family. Moreover, the performance of the ESM2-33 is comparable with ESM2-36 and ESM2-48, but the latter two models have more parameters, leading to a significant increase in runtime. Specifically, the ESM2-33 model consists of $\sim $650 million parameters, while the ESM2-36 and ESM2-48 models contain 3 billion and 15 billion parameters, respectively. Therefore, we chose ESM2-33 to embed the proteins.

We define $d_{e}$ as the dimension of per-residue embedding and impose a maximum limit of 1024 residues for each protein sequence, which aligns with the default setting of the ESM2 model. By applying the ESM2 embedding to the protein sequences, we generate an embedding matrix $X_{1}$ with dimensions of $1024 \times d_{e}$ for each protein shown in Fig. 2B. In the ESM2-33 model, the default value of $d_{e}$ is 1280. Subsequently, we pass $X_{1}$ through a fully connected (FC) layer, resulting in a 1D feature set denoted as $X_{2}$. We considered the mean and max pooling methods for protein embedding. The comparison in the “Ablation Study” of Supplementary Material shows that protein embeddings generated using the fully connected (FC) layer achieve better performance.

### Learning the relationship of context proteins using Transformer

As words and sentences in human language derive meaning through their context and relationships with other linguistic elements, proteins can also be better understood by considering their interactions, dependencies, and roles within the genome. Therefore, we annotate the protein functions by considering the context neighbors. This goal is achieved by preparing the context protein embedding and learning the relationships among proteins within the same genome. The sequential steps are illustrated in Fig. 2C.

To obtain the context protein embedding, first, we treat each protein as a token and contigs can be seen as sentences composed of multiple tokens. Then, we combine the embeddings of each protein into a single embedding with dimensions of $n \times d_{e}$. This integration process considers the order in which the proteins appear in the contigs and allows us to preserve the contextual relationships among the proteins within the same genomic context.

We use position embeddings to incorporate positional information. This component generates an embedding vector for each protein index, encoding its relative position within the sequence. The final output $X_{3}$ of the embedding layer is obtained by summing the context protein embedding and position embedding results, resulting in a comprehensive representation of each protein in the sequence.

After embedding the context proteins into an $n \times d_{e}$ matrix, we introduce a crucial component in our architecture: the self-attention layer. This layer plays a vital role in learning intricate connections between proteins. To perform self-attention computations in Equation (1), we transform the input matrix into three separate matrices: Query (Q), Key (K), and Value (V) through three independent FC layers. The $n \times n$ attention matrix is computed by multiplying the Q and K, representing the strength of protein associations. To prevent excessive values, we scale the attention matrix by dividing it by the square root of the dimension of matrix K (denoted as $\sqrt{d_{k}}$). Next, we normalize the attention matrix using the softmax function, assigning weights to protein pairs to indicate their relative importance. Finally, we score the proteins in the sequence by multiplying the V with the weight matrix.

In order to collectively focus on information stemming from diverse representation subspaces, we employ a multi-head mechanism in Equation (2), where each head represents a separate self-attention layer. Computation is performed in parallel across all heads, and then the concatenated head is input into an FC layer as shown in Equation (3), $W^{M} \in \mathbb{R}^{\text{1280} \times \text{ 1280 }}$. Following the multi-head attention block, the resulting output $X_{4}$ is passed through a feed-forward layer. 


(1) \begin{align*} & \text{ Attention }(Q, K, V)=\operatorname{softmax}\left(\frac{Q K^{T}}{\sqrt{d_{k}}}\right) V, \end{align*}

(2) \begin{align*} & \text{head}_{i}=\text{ Attention }\left(Q_{i}, K_{i}, V_{i}\right), \end{align*}

(3) \begin{align*} & X_{4}=FC( \operatorname{Contact}\left(\text{head}_{1}, \ldots, \text{head}_{n}\right), W^{M}). \end{align*}

### Predicting the GO terms

We formulate the protein function annotation task as a multi-label binary classification task. The goal is to assign a probability to each GO term, indicating the likelihood of the protein being associated with that specific function. The feed-forward layer result $X_{5}$ is input into a fully connected layer with the sigmoid activation function and the output is an m-dimensional vector, where $m$ represents the number of GO terms shown in Equation (4). 


(4) \begin{align*}& Y=\operatorname{sigmoid}\left(W \cdot X_{5}+b\right).\end{align*}

During training, the model is optimized by minimizing the binary cross-entropy loss. This loss function in Equation (5) is commonly used in binary classification tasks to measure the difference between predicted probabilities and actual labels. In addition, we train three GO prediction models on BP, CC, and MF separately. 


(5) \begin{align*}& \mathcal{L}=-\frac{1}{N} \sum_{i=1}^{N} \sum_{j=1}^{|\mathrm{GO}|} y_{i j} \log \left(\hat{y}_{i j}\right)\end{align*}

### Integrating GOPhage with alignment-based method

Considering that proteins with significant alignment usually have high-precision GO prediction results [13, 23], we introduce a hybrid mode named $\mathrm{GOPhage}^{+}$ by incorporating DiamondScore into the GOPhage to enhance the predictive capabilities for phage protein annotations. 


(6) \begin{align*}& \mathrm{S}_{\mathrm{GOPhage}^{+}}(i) = \beta \cdot \mathrm{S}_{\mathrm{GOPhage}}(i)+(1-\beta) \mathrm{S}_{\mathrm{DiamondScore}}(i),\end{align*}

where $\mathrm{S}_{\mathrm{GOPhage}^{+}}(i)$ is the confidence score of $\mathrm{GOPhage}^{+}$ for protein $i$, and $\mathrm{S}_{\mathrm{GOPhage}}(i)$ and $\mathrm{S}_{\mathrm{DiamondScore}}(i)$ are confidence scores of GOPhage and DiamondScore, respectively. The weight parameter $\beta $ is fine-tuned based on the validation dataset. Considering the hierarchical nature of GO terms, it is logical to maintain the predicted probability of a given GO term at least equal to or higher than that of all its child terms. We evaluate the effect of up-propagation on performance. The results in the section “Test the Effect of Up-Propagation” in the Supplementary Material show that, despite not explicitly incorporating the topology of GO terms into our model, the model is capable of implicitly learning this hierarchical structure from the training dataset.

## Results

### Metrics

We evaluate the performance of GOPhage following previous work. Specifically, we present two sets of metrics, corresponding to the prediction accuracy of protein-centric and GO term-centric evaluation, which are used in the Critical Assessment of Functional Annotation (CAFA) competitions. The protein-centric evaluation focuses on determining the function prediction accuracy, whereas the term-centric evaluation aims to examine whether the model can correctly identify proteins associated with a particular functional term [24]. The latter can provide the performance of different function terms.

First, we introduce protein-centric metrics. Let $P_{i}(t)$ be the set of GO terms for a protein $i$ returned by the model under the score cutoff $t$, while $T_{i}$ represents the true GO term set for protein $i$. Then recall and precision for each protein $i$ with threshold $t$ are calculated in Equations (7) and (8). To calculate the average recall and precision on all proteins, we define $n$ as the total number of proteins and $n_{t}$ as the number of proteins that have at least one predicted GO term when the threshold is $t$. The equations are shown in Equations (9) and 10, respectively. We record the F1-score calculated for each threshold $t$, ranging from 0 to 1, and obtain the maximum F1-score as $F_{\max }$ shown in Eqn. 11. To compute AUPR, the prediction scores of proteins are concatenated and input into the *scikit-learn* Python package. 


(7) \begin{align*} & recall_{i}(t)=\frac{ | P_{i}(t) \cap T_{i} |}{|T_{i}|} \end{align*}

(8) \begin{align*} & pre_{i}(t)=\frac{|P_{i}(t) \cap T_{i}|}{|P_{i}(t)|} \end{align*}

(9) \begin{align*} & AvgRecall(t)=\frac{1}{n} \cdot \sum_{i=1}^{n} recall_{i}(t) \end{align*}

(10) \begin{align*} & AvgPre(t)=\frac{1}{n_{t}} \cdot \sum_{i=1}^{m(t)} p re_{i}(t) \end{align*}

(11) \begin{align*} & F_{\max} =\max_{t}\left\{\frac{2 \cdot \operatorname{AvgPre}(t) \cdot \operatorname{AvgRecall}(t)}{\operatorname{AvgPre}(t)+\operatorname{AvgRecall}(t)}\right\} \end{align*}

Then, we present the term-centric evaluation. To calculate the term-centric $F_{\max }$, we follow a three-step process. First, we calculate the precision and recall for GO term $l$ under threshold $t$, as defined in Equations (12) and (13). In the second step, we calculate $F_{\max }(l)$, which is the maximum F1-score for label $l$ under different score cutoffs (Equation 14). Finally, we average these $F_{\max }(l)$ values across all GO labels to obtain the final $F_{\max }$, as shown in Equation (15). The AUPR for each label is calculated and averaged to obtain the final AUPR. 


(12) \begin{align*} & pre_{l}(t)=\frac{\sum_{i} I\left(l \in P_{i}(t) \wedge l \in T_{i}\right)}{\sum_{i} I\left(l \in P_{i}(t)\right)} \end{align*}

(13) \begin{align*} & recall_{l}(t)=\frac{\sum_{i} I\left(l \in P_{i}(t) \wedge l \in T_{i}\right)}{\sum_{i} I\left(l \in T_{i}\right)} \end{align*}

(14) \begin{align*} & F_{\max } (l)=\max_{t}\left\{\frac{2 \cdot pre_{l}(t) \cdot recall_{l}(t)}{p re_{l}(t)+recall_{l}(t)}\right\} \end{align*}

(15) \begin{align*} & F_{\text{max }}=\sum_{l=0}^{m} F_{\max }(l) \end{align*}

### Dataset

We downloaded the reference genomes and proteins under the Caudoviricetes class from the NCBI RefSeq database. Due to the lack of GO terms in the Refseq database, we mapped the protein accessions into UniProt database [25] using the “ID mapping” tool and retrieved annotations.

To ensure an adequate number of labeled proteins for training, we labeled the proteins with no GO terms using the Prokaryotic Virus Remote Homologous Groups (PHROG) database [26] based on HHsuite tool [27]. The database contains 38 880 PHROGs, encompassing 868 340 proteins derived from complete genomes of viruses infecting bacteria or archaea. Moreover, we saved the hits that demonstrated a probability of the template being homologous to query sequences exceeding 80%, ensuring the reliability and high confidence of the matches between the phage proteins and the entries in the PHROG database. Although we used the pairwise alignment to extend the dataset, the proteins with significant alignments were only 15.51%. The remaining 63.64% proteins still lacked annotations, which further demonstrated the necessity and importance of developing an effective phage protein annotation tool.

Because of the requirement for rich protein contextual information, we selectively focused on proteins from genera with high annotation rates. The annotation rate for each genome is calculated below. 


(16) \begin{align*}& \text{ Annotation Rate }=\frac{\#\text{proteins with annotation }}{\#\text{total proteins }}\end{align*}

Then, we computed the average annotation rate of the complete genomes in each genus. Proteins from genera where the annotation rates exceeded 30% for the BP and MF categories and 20% for the CC category are included. It was important to note that the number of proteins annotated by CC terms was relatively smaller than BP and MF. Therefore, we set a lower threshold for CC to ensure to inclusion of more genera. By setting these thresholds, we aimed to focus on genera with more comprehensive annotation information. The annotation rates for all genera can be found in the Supplementary material. We compared GOPhage with other tools using two datasets, with the details outlined below.

- High annotation rate dataset. All genera are sorted based on their annotation rates. We excluded single-genome genera, as they were insufficient for training purposes, resulting in a total of 598 genera. Utilizing the thresholds mentioned above, we retained the top 62 genera, 59 genera, and 203 genera for BP, CC, and MF, respectively.
- Leave-genus-out dataset. To thoroughly assess the generalizability of GOPhage, we selected an additional 10 genera that were not included in the training dataset. These genera were chosen based on sorted annotation rates, specifically those ranked 63–72 for BP, 60–69 for CC, and 204–213 for MF.

To minimize the similarity between the training and test/validation datasets, we implement the following steps for partitioning the high annotation rate dataset:

- The proteins obtained from the selected genera are aligned against all using the DIAMOND BLASTP [28] with a default e-value threshold of 0.001. The alignment scores among proteins are used to build a graph. Then Markov clustering algorithm (MCL) [29] is applied to cluster the protein graph, which is a fast and unsupervised method.
- We randomly select clusters and include all proteins within those clusters in the training dataset until the cumulative size exceeds 80% of the total dataset. The remaining proteins are placed in an independent dataset for evaluation purposes.
- Finally, we randomly divide the independent dataset into two equal-sized parts while ensuring an even distribution of proteins for each GO term label.

The GO labels are obtained by propagating all ancestors based on the “is_a” relationship in the tree. Then, we calculate the number of proteins annotated by each GO term and filter out terms with fewer than 200 annotated proteins in the training dataset. We follow the standard practice of CAFA assessment and exclude the root terms. The final protein and label number for three ontologies are shown in Supplementary Table 1.

To enhance the user experience, we provide two variant versions of GOPhage. The first version, named $\mathrm{GOPhage}_{\mathrm{LARGE}}$, is based on ESM2-33 and offers superior performance at the cost of increased computational resources and runtime. The second variant $\mathrm{GOPhage}_{\mathrm{BASE}}$ utilizes ESM2-12, providing a lightweight alternative with reduced computational demands. Specifically, we conducted tests on the prediction runtime for 1000 proteins. The results indicate that $\mathrm{GOPhage}_{\mathrm{LARGE+}}$ takes 13 min to annotate proteins across three ontologies, while $\mathrm{GOPhage}_{\mathrm{BASE+}}$ requires 4.84 min. Moreover, the parameter count for $\mathrm{GOPhage}_{\mathrm{LARGE}}$ is approximately seven times higher than that of $\mathrm{GOPhage}_{\mathrm{BASE}}$, as outlined in Supplementary Table 2.

### GOPhage outperforms the state-of-the-art predictors

In this experiment, we compared GOPhage with four tools: DiamondScore [13], DeepGOCNN [13], DeepGOPlus [13], and PFresGO [15]. These tools are the most widely used pipelines for general protein function annotation and have been demonstrated as state-of-the-art predictors. The same training dataset was utilized for retaining the learning-based methods (DeePGOCNN, DeepGOPlus, and PFresGO) or constructing the database for the alignment-based methods (DiamondScore). The performance evaluation was then carried out using the same test dataset, which ensured a fair and comparable assessment for all methods.

The performance based on term-centric is presented in Table. 2, while the results obtained from protein-centric evaluation can be found in Supplementary Table 3. $\mathrm{GOPhage}^{+}$ outperforms the second-best method, regarding both AUPR and Fmax scores with notable improvements across all three categories, specifically, the improvements of 9.94%, 6.50%, and 10.67% in AUPR and 6.49%, 4.67%, and 6.65% in Fmax scores for BP, CC, and MF, respectively.

**Table 2:** Performance comparison of GOPhage/GOPhage^+^ and state-of-the-art methods for protein function prediction based on term-centric evaluation in high annotation rate dataset.

	BP		CC		MF	
	AUPR	Fmax	AUPR	Fmax	AUPR	Fmax
DiamondScore	0.7225	0.6710	0.7552	0.6269	0.6557	0.6446
DeepGOCNN	0.6222	0.6380	0.6353	0.6455	0.4348	0.4590
DeepGOPlus	0.7279	0.7349	0.7623	0.7489	0.6304	0.6590
PFresGO	0.7642	0.7692	0.8232	0.8026	0.7210	0.7430
DeepGO-SE	0.7311	0.7500	0.8500	0.8276	0.7757	0.7869
$\mathrm{GOPhage}_{\mathrm{BASE}}$	0.7946	0.7814	0.8636	0.8108	0.7368	0.7505
$\mathrm{GOPhage}_{\mathrm{LARGE}}$	0.8382	0.8115	0.8664	0.8399	0.8125	0.7974
$\mathrm{GOPhage}^{+}_{\mathrm{BASE}}$	0.8595	0.8263	**0.8882**	0.8410	0.7804	0.7870
$\mathrm{GOPhage}^{+}_{\mathrm{LARGE}}$	**0.8636**	**0.8341**	0.8783	**0.8493**	**0.8277**	**0.8095**

Comparing $\mathrm{GOPhage}_{\mathrm{BASE}}$ and $\mathrm{GOPhage}_{\mathrm{LARGE}}$, the results reveal that using a larger protein foundation model has a better performance. The most significant improvement is observed in the MF category, with a notable increase of 7.57% in AUPR and 4.69% in Fmax. Additionally, integrating DiamondScore with GOPhage through hybrid approaches can further improve the performance in protein function prediction. Comparing $\mathrm{GOPhage}$ and $\mathrm{GOPhage}^{+}$, the BP category exhibits the highest improvement of 6.49% and 4.49% in AUPR and Fmax for $\mathrm{GOPhage}^{+}_{\mathrm{BASE}}$ and 2.54% and 2.26% in AUPR and Fmax for $\mathrm{GOPhage}^{+}_{\mathrm{LARGE}}$.

Taken together, utilizing a deeper foundation model and integrating homologous search methods can help GOPhage achieve the best performance in protein function prediction.

### GOPhage improves annotation of proteins by utilizing the contextual information

In this section, we designed two experiments to evaluate how contextual proteins impact function prediction. In the first experiment, we compare two different usages of the protein embeddings from the foundation model: (1) using the per-residue embedding of a single protein as input, and (2) using joint embeddings of multiple proteins with genomic context. A model named “Trans” is designed for a single protein input, which uses the amino acids as tokens and utilizes the Transformer to learn the relationship of residues. A detailed description of the methods is in the Supplementary File. For the multiple protein input, the GOPhage is used to learn the protein associations and predict the GO terms. In the second experiment, we compare the performance of GOPhage in different protein context sizes by gradually increasing the number of context proteins. This step-by-step analysis provides insights into how the augmentation of context information influences the model’s performance.

**Figure 3:**
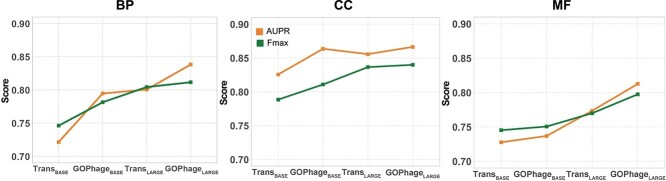
Performance comparison of including versus excluding contextual proteins across three ontologies, evaluated using AUPR and Fmax metrics for term-centric analysis.

Figure 3 shows the results for the first experiment. Based on the ESM2-12 model, a comparison between $\mathrm{Trans}_{\mathrm{BASE}}$ and $\mathrm{GOPhage}_{\mathrm{BASE}}$ reveals that BP and CC exhibit improvements of 7.3% and 3.8% in AUPR, respectively. Additionally, Fmax shows enhancements of 3.52% and 2.23% for BP and CC, respectively. Similarly, based on the ESM2-33 model, a comparison between $\mathrm{Trans}_{\mathrm{LARGE}}$ and $\mathrm{GOPhage}_{\mathrm{LARGE}}$ indicates that MF demonstrates the most significant improvement, with increases of 3.90% and 2.75% in AUPR and Fmax, respectively.

In the second experiment, we fed sentences with an increasing number of proteins into our model to show the impact of different contextual information. This involves creating three datasets:

- **Length>2 dataset**. We select protein sentences whose length is two or greater. Our goal is to preserve the original context information for our subsequent annotation.
- **Length=1 dataset**. From the “Length>2” dataset, we extract individual proteins by dividing the selected protein sentences. This dataset ensures that each sentence includes only one protein, thereby removing the contextual information.
- **Length=2 dataset**. Taking the protein sentences from the “Length>2” dataset, we divide them into pairs of two proteins with one overlap. Each sentence contains two proteins, representing an increase of one context protein compared with the “Length=1” dataset.

**Figure 4:**
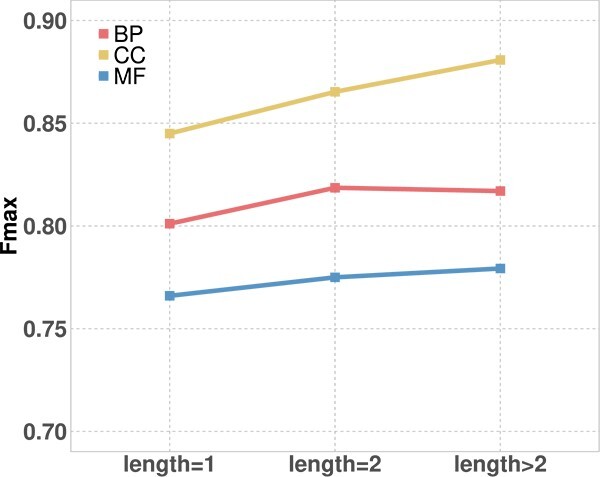
The performance on different numbers of context proteins from “length = 1” to “length >2” based on the Fmax of protein-centric.

By inputting three datasets containing varying levels of contextual information into our model, we observed notable trends in performance, as illustrated in Fig. 4. The results indicate a consistent pattern of performance enhancement as the number of context proteins is progressively augmented. As more contextual information is provided, the model better understands the relationships and interactions between proteins, resulting in improved predictions of protein functions. These findings emphasize the importance of considering contextual proteins and their impact on protein function prediction tasks.

### GOPhage shows superior performance in annotating novel proteins

**Figure 5:**
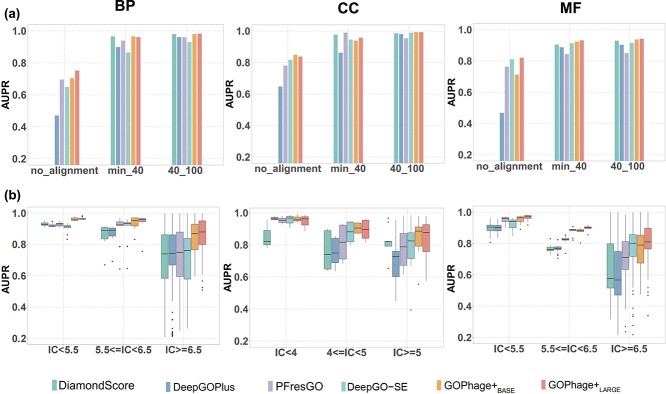
Performance comparisons among methods across three ontologies. (a) displays AUPR for protein-centric analysis across diverse sequence identity groups, and (b) shows AUPR for term-centric analysis across groups with increasing IC values.

In this section, we evaluate GOPhage’s predictive capability with different levels of sequence identity. The test dataset was partitioned into three distinct groups based on alignment with the training data: “no-alignment,” “min-40%,” and “40%–100%.” As shown in Fig. 5a, the AUPR of all methods improved with increased sequence identity for all three GO categories. For the high-similarity dataset, the alignment-based method exhibits excellent performance, and $\mathrm{GOPhage}^{+}$ demonstrates comparable results in three ontologies. It suggests that both methods can effectively predict protein functions when the dataset aligns well with the training dataset. However, for the dataset that has no alignment with the training dataset, $\mathrm{GOPhage}^{+}$ stands out with impressive AUPR. Specifically, $\mathrm{GOPhage}^{+}$ achieves AUPR values of 0.7524, 0.8478, and 0.8210 for the BP, CC, and MF. These values represent improvements of 5.68%, 6.78%, and 5.75% compared with the performance of the second-best method. The term-centric results are shown with a similar trend in Supplementary Fig. 1a. Additionally, the percentage of no-alignment proteins accounts for 27.93%, 55.70%, and 27.62% of the test dataset for BP, CC, and MF, respectively. These results highlight the robustness and effectiveness of $\mathrm{GOPhage}^{+}$ in predicting protein functions, especially for low-similarity proteins.

We continue to analyze the impact of contextual protein information on different level-similarity groups. The results are shown in the Supplementary Fig. 1b. Focusing on the no-alignments dataset, both $\mathrm{GOPhage}_{\mathrm{BASE}}$ and $\mathrm{GOPhage}_{\mathrm{LARGE}}$ demonstrate improvements compared with their respective counterparts. On one hand, $\mathrm{GOPhage}_{\mathrm{BASE}}$ shows performance gains of 10.18%, 6.5%, and 1.11% for BP, CC, and MF categories, respectively. On the other hand, $\mathrm{GOPhage}_{\mathrm{LARGE}}$ exhibits improvements of 5.33%, 3.87%, and 7.91% for BP, CC, and MF categories, respectively.

### GOPhage enhances annotation on minority-class GO terms

To examine GOPhage’s ability on GO terms of different popularities, we split them into three groups based on the information content (IC) of GO shown in Equation (17). ${f}\left (l\right )$ is the frequency of the GO term $l$ in the training dataset. Higher IC values mean fewer proteins annotated by the GO term labels. 


(17) \begin{align*}& \mathrm{IC}\left(l\right)=-\log_{2} f\left(l\right).\end{align*}

The experiment results in Fig. 5b demonstrate that all methods consistently performed well in the majority labels of GO terms. However, $\mathrm{GOPhage}^{+}$ demonstrates a distinct advantage in predicting minority GO terms, surpassing the other methods and achieving the highest performance across all three ontologies. Specifically, $\mathrm{GOPhage}^{+}$ achieves medium AUPR of 0.8801, 0.9043, and 0.8105 for BP, CC, and MF in the smallest GO terms group, respectively. This indicates that even for infrequently occurring GO terms, $\mathrm{GOPhage}^{+}$ can make an accurate prediction.

We also further investigate the impact of the context proteins on the different GO terms. The results are shown in Supplementary Fig. 1c. Focusing on the smallest GO terms group, both $\mathrm{GOPhage}_{\mathrm{BASE}}$ and $\mathrm{GOPhage}_{\mathrm{LARGE}}$ demonstrate improvements in performance. For the BP and CC ontologies, $\mathrm{GOPhage}_{\mathrm{BASE}}$ shows performance gains of 4.5% and 3.55% in AUPR, respectively. Moreover, it achieves comparable results for MF. In addition, $\mathrm{GOPhage}_{\mathrm{LARGE}}$ exhibits improvements of 3.71%, 2.29%, and 2.80% for BP, CC, and MF categories, respectively. These results highlight the benefits of incorporating context proteins in predicting fewer GO terms.

### GOPhage excels on unseen genera

To assess the generalizability of GOPhage, we evaluate its performance on the leave-genus-out dataset comprising 10 genera that are absent from the training dataset. The dataset consists of 1364, 832, and 9700 proteins for BP, CC, and MF, respectively. A comparative analysis of the term-centric evaluation with five other methods is presented in Table 3. Notably, $\mathrm{GOPhage}^{+}_{\mathrm{LARGE}}$ surpasses five methods in terms of both AUPR and Fmax scores across all three categories, achieving 0.8048, 0.7592, and 0.8052 in AUPR, and 0.7793, 0.7530, and 0.7890 in Fmax scores for BP, CC, and MF, respectively. The protein-centric performance is provided in Supplementary Table 4. $\mathrm{GOPhage}^{+}_{\mathrm{LARGE}}$ increases by 3.45%, 4.69%, and 2.46% in AUPR and 3.22%, 2.60%, and 2.05% in Fmax compared with the second-best method.

**Table 3:** Performance comparison of GOPhage/$\mathrm{GOPhage}^{+}$ and state-of-the-art methods on leave-genus-out dataset based on term-centric evaluation.

	BP		CC		MF	
	AUPR	Fmax	AUPR	Fmax	AUPR	Fmax
DiamondScore	0.7042	0.6183	0.7313	0.6439	0.7146	0.7229
DeepGOCNN	0.5136	0.5265	0.4035	0.4901	0.5337	0.5645
DeepGOPlus	0.6792	0.6700	0.6384	0.6689	0.6896	0.7287
PFresGO	0.7532	0.7450	0.7183	0.7117	0.7326	0.7493
DeepGO-SE	0.7079	0.7214	0.7186	0.7443	0.7628	0.7687
$\mathrm{GOPhage}_{\mathrm{BASE}}$	0.6660	0.6589	0.5982	0.6139	0.7447	0.7561
$\mathrm{GOPhage}_{\mathrm{LARGE}}$	0.7332	0.7066	0.6978	0.7286	0.8023	0.7865
$\mathrm{GOPhage}^{+}_{\mathrm{BASE}}$	0.7746	0.7497	0.7046	0.7217	0.7796	0.7638
$\mathrm{GOPhage}^{+}_{\mathrm{LARGE}}$	**0.8048**	**0.7793**	**0.7592**	**0.7530**	**0.8052**	**0.7890**

### GOPhage aids in identifying proteins lacking homology

To showcase the utility of GOPhage in annotating proteins that lack homology search results, we explore its application in the analysis of phage’s holin proteins. The holin protein is a small membrane protein that plays a crucial role in lysing bacterial hosts by triggering the formation of pores that disrupt the host cell membrane [30]. It controls the release of phages and the completion of the lytic cycle, underscoring the significance of the intricate interplay between phages and their host organisms. However, according to the protein annotation of phages in the RefSeq database, over $448$ genera have no annotated holin proteins, indicating that holin proteins may be very diverse across different phages. In this experiment, we apply GOPhage to annotate possible holin proteins.

**Figure 6:**
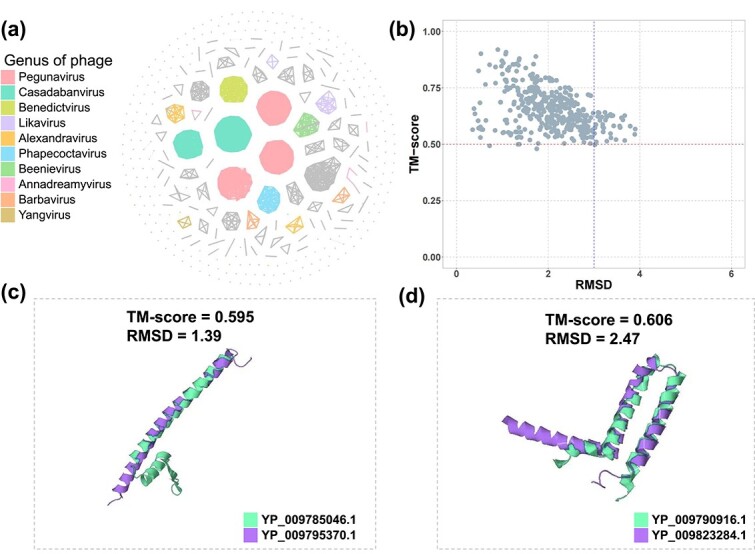
The analysis of the identified potential holin proteins. (a) and (b) show clusters within the top 10 phage genera and their structural similarities with known holin proteins, while (c) and (d) present 3D structures of identified holin proteins (YP_009795370.1 and YP_009823284.1) alongside database counterparts (YP_009785046.1 and YP_009790916.1).

According to statistical analysis of GO terms for the well-studied holin proteins from UniproKB, we manually selected six GO terms as their indicator. The details of selecting GO terms are shown in the Supplementary file. We input all proteins from $448$ genera into $\mathrm{GOPhage}^{+}$ and identified 688 potential holin proteins spanning $262$ genera. After identifying possible holins, we clustered them to analyze their relationship. To accomplish this, we aligned them all against all and selected alignment with identity and coverage larger than 90. Gephi [31] was used to represent the relationships among proteins visually. The results depicting the top 10 phage genera are shown in Fig. 6a. The genera of phage are from the RefSeq annotations. An evident observation is the high conservation of holin proteins within the same genus, mirroring a common pattern observed among known holin proteins in phage genomes.

In addition, we aligned them with the known holin proteins using BLASTP [32] with e-value 1e-5. A total of 590 proteins have no alignment, indicating the high diversity of holin proteins. Then, we searched the annotation of 688 proteins from the UniProtKB database. The automatic annotation pipeline provided by UniProt and designed to annotate uncharacterized protein sequences, known as ProtNLM [33], predicted that 335 of these proteins are holins. Additionally, 171 of the 688 proteins are labeled as uncharacterized proteins in UniProt, and 87 proteins are categorized as membrane proteins. Overall, these top three annotations account for 86.2% of the total proteins. To further examine the identified holin proteins without alignment, we employed ESMFold [20] to predict their 3D structures, which are very fast and can get comparable predictions with AlphaFold [34]. We found that despite having low sequence similarity, 590 identified holin proteins exhibit structural homology with the known holin proteins. The result is shown in Fig. 6b. The TM-score and the root mean square deviation (RMSD) value are calculated by the TM-align tool [35]. Figure 6c and d are visualizations of the two putative holin proteins identified by our tool. In conclusion, the experiments provide further evidence of the great potential of GOPhage as a valuable tool for viral protein annotation. In addition, the information and 3D structure of the 688 holins are available in the Supplementary data.

## Conclusion and discussion

In this work, we proposed a method named GOPhage/$\mathrm{GOPhage}^{+}$ for protein function annotation of phages. The major improvement in our approach can be attributed to utilizing the properties of phages and the foundation model. The Transformer model is used to learn the relationship of the genomic context proteins. Our experiments compared four methods including alignment-based and deep learning-based. They have shown that GOPhage can achieve the highest AUPR and $F_{max}$ across all three ontologies, especially on low-similarity and minority GO term labels. Furthermore, we investigated the impact of incorporating context proteins into the annotation process and observed that GOPhage exhibits significant improvements compared with using only individual proteins as input. Notably, GOPhage plays a crucial role in enabling the characterization of unannotated proteins, making it a valuable tool for biological discovery and in-depth investigations.

Given the increasing interest in engineering phages for various applications, it is important to consider the performance of GOPhage+ on modified or engineered phages. If the engineered changes do not significantly alter the overall genomic context of the modified phages, the performance should remain unaffected. However, for genomic arrangements that are not well represented in naturally occurring datasets, we recommend inputting individual proteins into our model for prediction, as this approach does not account for the influence of contextual proteins.

GOPhage is trained only on phages within the Caudoviricetes class, which accounts for 97% of the total reference genomes for prokaryotic viruses. The primary challenge for improving phage protein annotation is the limited number of proteins with available experimentally validated labels, which are inadequate to serve as the training dataset for deep learning models. Therefore, we utilize them solely as external test datasets and showcase the outcomes in Supplementary Tables 8 and 9, aiming to provide a reference for potential users. By including proteins with enriched GO terms, we can augment the pool of context proteins available for analysis. Furthermore, the incorporation of additional proteins will amplify the number of GO term labels and facilitate the annotation of phage proteins at a more specific and detailed level, which will provide valuable insights into the intricate functional characteristics of these proteins. In the future, incorporating additional features such as structure information derived from GO graphs and textual descriptions of proteins is a valuable direction for further improving the annotation process.

**Key Points**

- Inspired by the modular genomic structure of phage genomes, GOPhage is designed by utilizing the latest foundational model and the Transformer model to learn the contextual relationship of proteins.
- GOPhage demonstrates superior performance in annotating novel proteins that are commonly discovered in metagenomic sequencing, enhancing our understanding of phages.
- GOPhage can identify core functional proteins of phages, such as holins, from unannotated proteins. Notably, many of the identified potential holin proteins lack sequence similarity with known holins yet exhibit structural homology to them.

## Supplementary Material

Supplementary_Material_for_GOPhage_Protein_function_annotation_bbaf014

supplementary_1213_bbaf014

## Data Availability

GOPhage is implemented in Python, which can be downloaded from https://github.com/jiaojiaoguan/GOPhage.
