# Supplementary material for: GOPhage: protein function annotation for bacteriophages by integrating the genomic context
Source: Brief Bioinform. 2025 Jan 22;26(1):bbaf014. doi: 10.1093/bib/bbaf014 (PMC11751364; doi:10.1093/bib/bbaf014)
Supplement: Supplementary_Material_for_GOPhage_Protein_function_annotation_bbaf014 [file supplementary_material_for_gophage_protein_function_annotation_bbaf014.pdf]

Supplementary Material for

GOPhage: Protein function annotation for bacteriophages by integrating the genomic context

The architecture of the Trans model

To demonstrate the impact of contextual information on protein function annotation, we developed a model named 'Trans'. The architecture of 'Trans' comprises three main steps. Initially, a protein with m residues is fed into the ESM2 foundation model, generating per-residue embeddings of dimensions $m \times d_e$. Based on the setting of the GOPhage, the m is 1024 and the d_e is 1280. Subsequently, each amino acid in the protein sequence is treated as a token, with the m per-residue embeddings serving as word embeddings and inputted into the Transformer model. This model captures relationships among the residues. The learned features of each residue from the Transformer model are then passed through a Fully Connected (FC) layer for classification.

The number of the parameter of GOPhage models

We tabulated the parameter counts for two versions of the GOPhage models in Table 2. As we trained distinct models for the three ontologies, we calculated the parameters for each separately. Due to variations in the number of GO term labels and the length of input sentences across the three ontologies, the parameter counts differ. Notably, the number of parameters of GOPhage$_{\text{LARGE}}$ is 6.91, 7.06, and 6.85 times that of GOPhage$_{\text{BASE}}$ in the BP, CC, and MF ontologies, respectively. In addition, based on the performance, GOPhage$^{+}_{\text{BASE}}$ and GOPhage$^{+}_{\text{LARGE}}$ are recommended for users. However, in scenarios where computational resources are constrained, GOPhage$^{+}_{\text{BASE}}$ is the preferable choice.

Overall protein-centric performance of GOPhage

The experiment compared GOPhage and GOPhage^{+} with four other methods: DiamondScore [1], DeepGOCNN [1], DeepGOPlus [1], and PFresGO [2]. The results based on a protein-centric evaluation are presented in Table 3. Both GOPhage and GOPhage^{+} achieved the best performance compared to the other methods. Specifically, GOPhage^{+} exhibited remarkable improvements over the second-best method, with increases of 8.32%, 4.82%, and 10.24% in AUPR, and 2.31%, 1.28%, and 13.09% in Fmax for BP, CC, and MF, respectively. Comparing the two versions of GOPhage, the performance of GOPhage$_{\text{LARGE}}$ surpassed that of GOPhage$_{\text{BASE}}$, highlighting the advantages of deeper foundation models. In particular, GOPhage$_{\text{LARGE}}$ demonstrated improvements of 3.65%, 0.47%, and 6.28% in Fmax for BP, CC, and MF, respectively. Furthermore, integrating a similarity-based method further enhanced the performance. GOPhage$^{+}_{\text{BASE}}$ improved AUPR by 4.63%, 1.16%, and 4.68% for BP, CC, and MF, respectively. Similarly, GOPhage$^{+}_{\text{LARGE}}$ showed improvements of 2.21%, 1.04%, and 0.79% in AUPR for BP, CC, and MF, respectively. Overall, these findings demonstrate that GOPhage and GOPhage^{+} outperformed the other methods by integrating deeper foundation models and similarity-based approaches.

Table 1. The number of the proteins and the Go term labels in training, validation, and test dataset.

	Train	Test	Val	Term
CC	3170	393	394	23
BP	4974	834	755	126
MF	30301	4012	3835	165

Table 2. The number of the parameter for two versions of the GOPhage model.

	BP	CC	MF
GOPhage$_{\text{BASE}}$	1,448,831	1,399,288	1,467,590
GOPhage$_{\text{LARGE}}$	10,005,631	9,873,688	10,055,590

Define the criteria for selecting the GO terms for holin proteins

We retrieved well-studied virus holin proteins with an annotation score greater than 2 from the UniProtKB database, resulting in a total of 1321 proteins. In the Cellular Component (CC) ontology, 98.33% of these proteins are associated with the terms GO:0016020 (membrane) and GO:0020002 (host membrane). In the Molecular Function (MF) ontology, 98.03% of the proteins possess the term GO:0140911 (pore-forming activity). In the Biological Process (BP) ontology, 98.33% of the proteins exhibit GO:0031640 (killing of cells of another organism), 69.27% show GO:0019076 (viral release from host cell), and 68.36% have GO:0044659 (viral release from host cell by cytolysis). We identify the holin proteins if the gene ontology annotation includes GO:0016020, GO:0140911, and one of the three BP GO terms. Applying these criteria, our method GOPhage^{+} can identify 688 proteins as potential holin proteins.

Ablation Study

This study investigates the impact of different protein embeddings on performance. GOPhage employs the FC layer to transform per-residue embeddings from a shape of $L \times d$ to per-protein embeddings with a shape of $1 \times d$. We compare the mean and max pooling methods, presenting the results in Table 5 and Table 6. The findings reveal that max-pooling performs the poorest across all three ontologies. When assessing the output from a protein-centric perspective, the results obtained using the FC layer are comparable or slightly lower (-0.06% to -2.06%) than those from the mean pooling methods. However, when examining the term-centric outcomes, the FC methods demonstrate a substantial improvement of 4.26% to 10.30% over mean pooling. Consequently, based on the comprehensive analysis, we opt for the FC methods to generate the per-protein embeddings.

Table 3. The performance of all methods on the high annotation rate dataset based on the protein-centric evaluation.

	BP		CC		MF	
	AUPR	Fmax	AUPR	Fmax	AUPR	Fmax
DiamondScore	0.796	0.6802	0.7801	0.4194	0.7462	0.6602
DeepGOCNN	0.7281	0.6718	0.7938	0.7731	0.6215	0.5897
DeepGOPLUS	0.8264	0.7752	0.8539	0.8163	0.7770	0.7666
PFresGO	0.8387	0.8486	0.8866	0.8577	0.8054	0.7310
DeepGO-SE	0.8306	0.8195	0.9192	0.8748	0.8832	0.8413
$\text{GOPhage}_{\text{BASE}}$	0.8688	0.806	0.9232	0.8638	0.8354	0.7897
$\text{GOPhage}_{\text{LARGE}}$	0.8998	0.8425	0.9186	0.8685	0.8999	0.8525
$\text{GOPhage}^{+}_{\text{BASE}}$	0.9151	0.8614	**0.9348**	0.8704	0.8822	0.8397
$\text{GOPhage}^{+}_{\text{LARGE}}$	**0.9219**	**0.8717**	0.9290	**0.8705**	**0.9078**	**0.8619**

Table 4. The performance of all methods on leave-genus-out dataset based on the protein-centric evaluation.

	BP		CC		MF	
	AUPR	Fmax	AUPR	Fmax	AUPR	Fmax
DiamondScore	0.6910	0.4376	0.6276	0.3083	0.8428	0.7966
DeepGOCNN	0.6188	0.6201	0.7190	0.7470	0.7363	0.6818
DeepGOPLUS	0.7293	0.7022	0.7925	0.8034	0.8347	0.8221
PFresGO	0.8316	0.7749	0.8128	0.8207	0.8731	0.8592
DeepGO-SE	0.7642	0.7370	0.8679	0.8789	0.8916	0.8686
$\text{GOPhage}_{\text{BASE}}$	0.7578	0.6872	0.8594	0.8594	0.8690	0.8150
$\text{GOPhage}_{\text{LARGE}}$	0.8196	0.7452	0.8951	0.8952	0.9118	0.8645
$\text{GOPhage}^{+}_{\text{BASE}}$	0.8421	0.7665	0.8984	0.8902	0.9055	0.8786
$\text{GOPhage}^{+}_{\text{LARGE}}$	**0.8661**	**0.8071**	**0.9148**	**0.9049**	**0.9162**	**0.8891**

Table 5. Comparison of the embedding using mean pooling, max pooling, and FC layer based on the ESM2-12 foundation model. The numbers in brackets represent the value of increase or decrease compared to the result of the mean pooling.

		BP	CC	MF
		Fmax	Fmax	Fmax
Term-centric	Mean	0.7632	0.7993	0.7028
	Max	0.4045	0.7112	0.4630
	FC	**0.8060(+4.26%)**	**0.8638(+6.45%)**	**0.7897(+8.69%)**
Protein-centric	Mean	0.7720	**0.8314**	0.7201
	Max	0.6493	0.7459	0.5564
	FC	**0.7814(+0.94%)**	0.8108(-2.06%)	**0.75050 (+1.51%)**

Test the effect of up-propagation

Taking into account the hierarchical nature of GO terms, it is logical to maintain the predicted probability of a given GO term equal to or higher than that of all its child terms. To assess the impact of up-propagation, we initially computed the average error rate for each protein without up-propagation. Specifically, we examined the predicted probabilities for each protein and compared the probabilities of child GO terms with their respective parent GO terms. An error was recorded when the probability of a child term exceeded that of its parent term. The error rate was then computed as the ratio of the number of errors to the total number of comparisons made within the GO terms hierarchy. The error rates are just 0.69%, 1.39%, and 0.43% for BP, CC, and MF. The low error rates suggest that, despite not explicitly incorporating the topology of GO terms into our model, the model is capable of implicitly learning this hierarchical structure from the training dataset. To further refine our predictions, we adjusted any instances where the probability of a child term was greater than that of its parent term by setting the child's probability equal to the parent's probability. The results of this adjustment, both protein-centric and term-centric, are also provided in Table 7. Upon comparison, it is evident that the adjusted results are comparable to the original results.

Performance on experimentally annotated proteins

To assess the performance of experimentally annotated proteins, we initially obtained the UniProt-GOA database from the following link: https://www.ebi.ac.uk/GOA/downloads. This database contains information such as protein names, taxon IDs, GO terms, and evidence codes. Within the subset of Caudoviricetes (Taxon ID: 2731619), there were a total of 1,522,526 proteins. To identify proteins with experimental validation, we focused on evidence codes including "EXP", "IDA", "IPI", "IMP", "IGI", "IEP", "TAS", "IC", "HTP", "HDA", "HMP", "HGI", and "HEP". Among these, we obtained 785 experimental annotations across 411 phage proteins. To ensure the test proteins are excluded from the training datasets of the existing tools, we examined the released dates of DeepGO-SE, GPSfun, and NetGO3. Subsequently, we filtered out proteins created before January 2022, retaining a total of 30 proteins. Since there is only one protein of CC ontology, our comparative analysis focused on the BP and MF ontologies. We directly utilized the released models available on GitHub for DeepGO-SE and PFresGO, employing the specific web servers of NetGO3 and GPSFun for conducting prediction tasks. The results of the protein-centric assessment are elaborated in Table 8. Furthermore, we compared our

Table 6. Comparison of the embedding using mean pooling, max pooling, and FC layer based on the ESM2-33 foundation model. The numbers in brackets represent the value of increase or decrease compared to the result of mean pooling.

		BP	CC	MF
		Fmax	Fmax	Fmax
Term-centric	Mean	0.7801	0.8099	0.7495
	Max	0.7156	0.7016	0.4866
	FC	**0.8425(+6.24%)**	**0.8685(+5.86%)**	**0.8525(+10.3%)**
Protein-centric	Mean	**0.8224**	**0.8405**	**0.7998**
	Max	0.7268	0.7900	0.5691
	FC	0.8115(-1.09%)	0.8399(-0.06%)	0.7974(-0.24%)

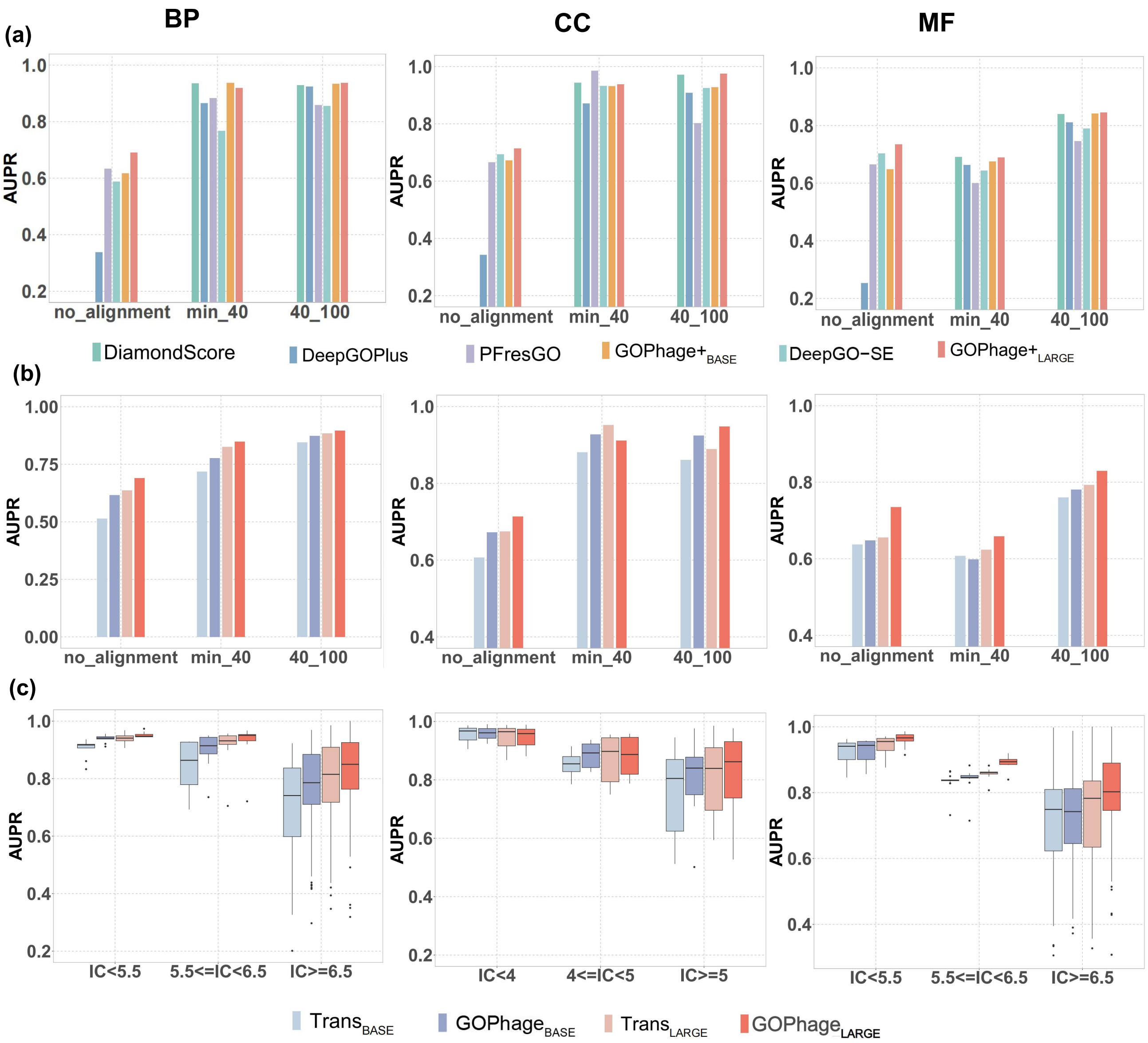

Fig. 1. The performance on different similarity datasets and different GO terms. (a) shows the AUPR of term-centric on six methods. (b) and (c) demonstrates the effect of the contextual proteins on three different identity groups and different labels on the protein function annotation task.

model with other existing methods across all 411 phage proteins mentioned by the reviewer without considering that some of them are likely part of the training data of different tools/webservers. The detailed results of the protein-centric evaluation can be found in Table 9 of the supplementary material. As some of the 411 phage proteins are encompassed within the training datasets of existing methods, we observe

Table 7. Comparison of the effect of up-propagation on three ontologies.

		BP		CC		MF	
		AUPR	Fmax	AUPR	Fmax	AUPR	Fmax
Term-centric	$\text{GOPhage}^{+}_{\text{LARGE}}$	0.8636	0.8341	0.8783	0.8493	0.8277	0.8095
	Adjust	0.8612	0.8355	0.8778	0.8483	0.8351	0.8091
Protein-centric	$\text{GOPhage}^{+}_{\text{LARGE}}$	0.9219	0.8717	0.9290	0.8705	0.9078	0.8619
	Adjust	0.9197	0.8716	0.9231	0.8722	0.9037	0.8610

Table 8. The performance on 30 experimentally annotated proteins based on the protein-centric evaluation.

	BP		MF	
	AUPR	Fmax	AUPR	Fmax
PFresGO	0.0517	0.1658	0.2425	0.4125
DeepGO-SE	0.0811	0.1734	0.3610	0.5886
GPSFun	0.3383	0.5258	0.3983	0.5712
NetGO3	0.3276	0.5718	0.5869	**0.6644**
$\text{GOPhage}_{\text{LARGE}}+$	**0.6151**	**0.6848**	**0.6238**	0.6096

Table 9. The performance on 411 experimentally annotated proteins based on the protein-centric evaluation.

	BP		CC		MF	
	AUPR	Fmax	AUPR	Fmax	AUPR	Fmax
PFresGO	0.1575	0.2612	0.2437	0.3421	0.2099	0.3464
DeepGO-SE	0.2213	0.3068	0.3287	0.4325	0.5347	0.6008
GPSFun	0.3028	0.3811	0.3005	0.4850	0.6488	0.6988
NetGO3	0.4181	0.5422	0.4736	0.6262	**0.7572**	0.7576
$\text{GOPhage}_{\text{LARGE}}+$	**0.8490**	**0.8179**	**0.8613**	**0.9172**	0.7385	**0.7927**

a performance boost. In summary, GOPhage+ surpasses the current state-of-the-art methods in terms of performance.

References

1. Maxat Kulmanov and Robert Hoehndorf. DeepGOPlus: improved protein function prediction from sequence. *Bioinformatics*, 36(2):422–429, 07 2019.
2. Tong Pan, Chen Li, Yue Bi, Zhikang Wang, Robin B Gasser, Anthony W Purcell, Tatsuya Akutsu, Geoffrey I Webb, Seiya Imoto, and Jiangning Song. PFresGO: an attention mechanism-based deep-learning approach for protein annotation by integrating gene ontology inter-relationships. *Bioinformatics*, 39(3):btad094, 2023.
